# Psammocarcinoma of ovary with serous cystadenofibroma of contralateral ovary: a case report

**DOI:** 10.1186/1752-1947-3-9330

**Published:** 2009-12-15

**Authors:** Deepali Jain, L Akhila, Vibha Kawatra, Pallavi Aggarwal, Nita Khurana

**Affiliations:** 1 Department of Pathology, Maulana Azad Medical College, New Delhi, 110002, India

## Abstract

**Introduction:**

Psammocarcinoma of ovary is a rare serous neoplasm characterized by extensive formation of psammoma bodies, invasion of ovarian stroma, peritoneum or intraperitoneal viscera, and moderate cytological atypia. Extensive medlar search showed presence of only 28 cases of psammocarcinoma of ovary reported till date.

**Case presentation:**

We herein report a case of psammocarcinoma of ovary with serous cystadenofibroma of contralateral ovary in a 55 year old Asian Indian female.

**Conclusion:**

To the best of author's knowledge, ours is the rare case describing coexistence of this very rare malignant serous epithelial tumor with a benign serous cystadenofibroma of contralateral ovary.

## Introduction

Psammocarcinoma of ovary is a rare serous neoplasm. Diagnosis requires the psammoma bodies to be present in at least 75% of papillae which show a destructive invasion of the ovarian stroma. The neoplastic cells do not show more than moderate atypia. There should be no areas of solid proliferation of neoplastic cells. These tumors are associated with a better prognosis than conventional serous neoplasms with low recurrence following tumor resection [1]. Literature search showed presence of only 28 cases of psammocarcinoma of ovary reported till date (Table 1) [1-17]. This case will be another new one in the literature with serous cystadenofibroma of contralateral ovary and elevated levels of CA-125.

**Table 1:** Literature review of primary ovarian psammocarcinomas

Author	Year	No. of cases	Age	Clinical features	CA- 125 units/ml	FIGO stage	Surgery	Chemothearpy	Follow up	Remarks
Gilks et al [1]	1990	8	36 to 76 (mean of 57)	Abdominal pain	NA	III	TAH+ BSO (4 cases) LSO(2 cases) BSO(2 cases) Oment (3/8 cases)	Y (1 patient)	1-died 3- lost FU 4- free of disease	

Kelley et al [2]	1995	1	18	Abdominal pain	25	IIIC	TAH, BSO, oment,	Y	42 months NED	Adolescent

Pakos et al [3] (German)	1997	1	49	Mass in the lower abdomen		IA	BSO	N	NED	

Powell et al [4]	1998	1	59	Abdominal pain and increasing abdominal girth	118	IIIB	TAH, BSO, oment	N	12 months NED	Family history of epithelial cancer positive

Poggi et al [5]	1998	1	66	Abdominal Pain nausea vomiting	NA	IIIB	BSO, oment	N	Recurrence 18 months	Aggressive, with Cystadenofibromata

Cobellis et al [6]	2003	1	48	Referred for leiomyomata uteri	Normal	IIIA	TAH, BSO, oment	N	2 years NED	Omental and peritoneal implant

Giordano et al [7]	2005	1	66	Abdomino-pelvic mass	Elevated	IIIB	TAH, BSO, oment,	Y	NED after 1 year	Bilateral, omental nodule showed the features of invasive implant

Rattenmaier et al [8]	2005	1	70	Malaise and abdominal discomfort	25,000	NA	AH, BSO	N	Recovered	Bilateral with cysadenofibromata

Radin et al [9]	2005	1	60	Diffuse abdominal pain, bloating, diarrhea, and low back pain,	65.2	III	Laparotomy, tumor debulking	Y	NA	Aggressive

Vimplis et al[10]	2006	1	63	Abdominal discomfort and increasing abdominal girth	1,133	IIIB	BSO,, SH, oment,	Y	NED	

Hiromura et al [11]	2007	1	73	Lower abdominal distention and pain	464	IIIC	AH, BSO, and oment	Y	4 months stable	

Akbulut et al [12]	2007	1	67	Vaginal bleeding and abdomino-pelvic pain	175	IIIC	Debulking	Y	10 years with recurrent and metastatic disease	Aggressive

Pusiol et al [13]	2008	1	50	NA	NA	IIIB	Laparotomy	Y	10.5 years, free of disease.	Bilateral, psammoma bodies in cervical smear, Presence of psammocarcinoma in the tubaric lumen

Alanbay et al [14]	2009	2	41,50	Adnexal mass, pelvic pain	NA, 3,223	III	Surgery	Y	6 years free of disease, 2 months	

Tiro et al [15]	2009	1	58	Shortness of breath	175.5	NA	N	Y	NA	Implants in pleural cavity and pericardium

Chase et al [16]	2009	1	45	Subcutaneous nodule	NA	NA	Bilateral salpingo-oophrectomy	Tamoxifen	NA	Mediastinal, pulmonary, subcutaneous, and omental metastases

Poujade et al [17]	2009	4	19-67	NA	NA	NA	Y	Y except in one case	18-45 months NED, one case has persistent disease	

Current case	2009	1	50	Menorrhagia, abdominal discomfort and pain	995.4	I C	Total abdominal hysterectomy with bilateral salpingo oophorectomy	Y	6 months, free of disease	Contralteral cystadenofibroma

Abbreviations: NA- Not available; TAH - Total abdominal hysterectomy; BSO- Bilateral salpingo- oophorectomy; Oment- Omentectomy; AH- Abdominal hysterectomy; Y- Yes; N- No; FU- Follow up; NED- No evidence of disease, LSO - left salpingo-oophorectomy; SH- Subtotal hysterectomy

## Case presentation

**Figure 1:**
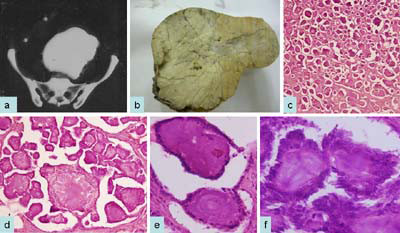
**Computed tomography scan shows calcified abdominopelvic mass (a); gross photograph shows gritty and firm tumor (b); on histological examination, tumor reveals extensive psammoma bodies which are surrounded by single layer of cytologically bland cuboidal or low columnar epithelium (c-f) H&E cx40; dx100; ex400; fx600**.

A 55-year-old Asian Indian female presented to gynecology clinic with complaints of menorrhagia, abdominal discomfort and pain. On Examination a mass was palpable in the lower abdomen which was non tender and fixed. Ultrasound (USG) examination revealed the presence of an abdomino-pelvic mass and ascites. Contrast-enhanced computed tomogram (CECT) revealed an abdominopelvic lobulated calcified mass measuring 17 × 15 × 16 cms (Fig 1a). Clinically, a possibility of calcified fibroid of uterus was suspected. However, cytologic examination of ascitic fluid was positive for malignant cells. On exploratory laparotomy, bilateral ovaries were enlarged with intact capsule. Total abdominal hysterectomy with bilateral salpingo-oophorectomy was performed. There were no retroperitoneal lymph nodes. No visible tumor nodules were identified on peritoneal surface. Grossly the left ovary was enlarged and measuring 24 × 20 × 20 cms. Outer surface was smooth and lobulated. Cut section was solid, grey white, gritty and granular with no visible necrosis (Fig 1b). The right ovary measured 7 × 6 × 2 cms. Cut section was solid and cystic with cysts ranging in size from 0.4 to 0.5 cm. The cysts were filled with clear fluid. No areas of hemorrhage or necrosis were seen in either of the two ovaries. Microscopically, sections from the left ovary revealed numerous psammoma bodies infiltrating the ovarian stroma and forming more than 95% of the tumor. At places these were lined by low cuboidal cells with minimal atypia (Fig 1c-f). No areas of necrosis, hemorrhage or increase mitosis were seen. Sections from the right ovary showed cysts lined by low cuboidal epithelium with prominence of fibroblastic stromal component. Thus a final diagnosis of psammocarcinoma of left ovary and serous cystadenofibroma of right ovary was made. Following the diagnosis, serum CA-125 was estimated which was elevated to 995.4 U/ml. Uterus showed a submucosal leiomyoma with proliferative endometrium on histology. Cervix and bilateral fallopian tubes were unremarkable grossly as well as microscopically. Patient received chemotherapy and kept on close follow up.

## Discussion

Psammocarcinoma of ovary is a rare serous neoplasm which can arise from both peritoneum and ovaries [1]. Extensive search showed presence of only 28 cases of psammocarcinoma of ovary reported till date [1-17] (Table 1). Age ranged from 18 to 76 years [1,2]. Many of these cases presented with abdominal discomfort and increasing abdominal girth.

This tumor can be misdiagnosed as other calcifying tumors of abdomino-pelvic region especially calcified leiomyomas, by radiological investigations, similar to our case. However if calcified abdomino-pelvic tumors are seen in association with an elevated CA-125 level, then a diagnosis of ovarian neoplasm can be made [7]. In most of the reported cases CA-125 levels were raised ranging from 65-25,000 units/ml [7,8]. Our patient had elevated levels of CA-125 just after the surgery.

Although most of the cases reported are unilateral, rarely these are bilateral [8,13].

All but one, cases reported in the literature had FIGO stage III tumors showing clinical behavior similar to that of borderline serous tumors [3]. Our case fits in to stage I C as patient had malignant ascitis, however no pelvic extension was seen.

Despite the apparent low malignant potential of this tumor, there remains a need for patient's follow up data as aggressive behavior has been described in the literature [12,15,16]. In the case reported by Poggi et al [5] no adjuvant therapy was given because of the supposed indolent behavior. However, recurrence of disease has been noted after eighteen months. Our patient received post operative adjuvant therapy and is on follow up.

Histopathologic differential diagnosis of psammocarcinoma involves other serous epithelial tumors. Some rare cystadenofibromas have mild cytologic atypia and may also exhibit psammoma bodies, but they are generally inconspicuous. In the present case we have seen cystadenofibroma in the contralateral ovary with an occasional psammoma body formation. Earlier, association of psammocarcinoma with cystadenofibroma has been documented [5,8]. Although these tumors show clinical behavior similar to that of borderline serous tumors, the presence of destructive stromal invasion excludes serous borderline tumors. Moderate cytologic atypia helps differentiating from high grade serous carcinomas. Psammocarcinomas differ from well-differentiated serous adenocarcinomas by numerous psammoma body formations.

Potential mechanisms responsible for the characteristic, extensive psammoma body formation include the accumulation of successive layers of calcium on single necrotic or degenerated tumor cells. One hypothesis states that they arise due to accumulation of hydroxyapatite in degenerating cells [18]. Psammoma bodies are described as multiple, discrete, laminated calcified bodies and are found in many neoplastic and non neoplastic lesions. Characteristic calcifications on radiological investigations and psammoma bodies on cervicovaginal smears should alert the clinician for this unusual tumor. Recently Pusiol et al [13] have reported a case of psammocarcinoma associated with presence of psammoma bodies in the cervicovaginal smears.

The molecular features of psammocarcinoma include mutations of a gene belong to the cancer related RAF family, that is BRAF [19].

Hiromura et al [11] described imaging features of psammocarcinoma including extensive minute calcifications on CT scan that were not detected on the abdominal x-ray. The tumor appears sandy and coarsely granular on enhanced T1-weighted MR images due to scattered clusters of psammomatous calcifications. Radin et al [9] have shown its high avidity for the Tc-99m MDP radiopharmaceutical.

Like with other serous epithelial tumors of the ovary, aggressive debulking surgery has been the initial treatment modality in nearly all cases. Postoperative therapies have included observation, tamoxifen and cytotoxic chemotherapy (generally using cyclophosphamide with cisplatin or carboplatin) [4].

## Conclusion

Psammocarcinomas are rare serous neoplasms. Due to the rare aggressive nature of the tumor, it is important to follow up the patient closely. Our case adds a new case of psammocarcinoma with contralateral cysadenofibroma in the literature.

## Consent

Written informed consent was obtained from the patient for publication of this case report and accompanying images. A copy of the written consent is available for review by the Editor-in-Chief of this journal.

## Competing interests

The authors declare that they have no competing interests.

## Authors' contributions

DJ, NK, VK, PA participated in conception of the idea, writing of the manuscript, and interpretation of histological assays. LA collected data. DJ participated in interpretation of biopsies, review of the literature, and writing of the manuscript.
